# Smart Shirt for Measuring Trunk Orientation

**DOI:** 10.3390/s22239090

**Published:** 2022-11-23

**Authors:** Abdella Ahmmed Simegnaw, Yetanawork Teyeme, Benny Malengier, Tamrat Tesfaye, Hundessa Daba, Kaledawit Esmelealem, Lieva Van Langenhove

**Affiliations:** 1 Centre for Textile Science, Engineering Department of Materials, Textiles and Chemical Engineering, Ghent University, 9000 Gent, Belgium; 2 Ethiopian Institute of Textile and Fashion Technology, Bahir Dar University, P.O. Box 1037, Bahir Dar 6000, Ethiopia; 3 Institute of Technology, School of Biomedical Engineering, Jimma University, Jimma P.O. Box 378, Ethiopia; 4 Bahir Dar Institute of Technology, Faculty of Computing, Computer Science, Bahir Dar University, P.O. Box 26, Bahir Dar 6000, Ethiopia

**Keywords:** gyroscope, accelerometer, smart garment, posture, trunk angle, textile sensor

## Abstract

Improper cycling posture is linked to a variety of spinal musculoskeletal diseases, including structural malformation of the spine and back discomfort. This paper presents a novel smart shirt integrated tri-axial gyroscope and accelerometer that can detect postural variation in terms of spinal curvature changes. To provide accurate feedback to the wearer and improve the wearer’s correct movement, the garment is able to recognize trunk body posture. The gyroscope/accelerometer was placed around the upper and mid trunk of the user to record tri-axial angular velocity data. The device can also be used to help determine the trunk bending angle and monitor body postures in order to improve optimal orientation and position. The garment enables continuous measurement in the field at high sample rates (50 Hz), and the sensor has a large measurement range (16 g, 2000°/s). As electronic components are non-washable, instead of encapsulating them, a detachable module was created. In this, magnets are embedded in the jersey, and allow the positioning and removal of the sensor. The test results show that the average trunk-bending angle was 21.5°, and 99 percent of the observed angle fell within the standard (ranging from 8° to 35°). The findings demonstrate the feasibility of employing the smart shirt sensor to estimate trunk motions in the field on a regular basis.

## 1. Introduction

Back pain affects a large group of people, with symptoms primarily affecting the lower back. Textile sensing systems provide many advantages when it comes to measuring body postures, most importantly, not being invasive and allowing measurement in a comfortable way. In addition to textile sensing systems, many novel systems have been developed recently that are convenient, portable, and may be worn anywhere at any time. Personal healthcare, rehabilitation, sports training, and performance monitoring can all benefit from textile posture movement sensing.

The incorporation of electronics [1] in technical textile has attracted more interest recently, particularly in developed countries. Numerous projects driven by the industry have already been launched. Varieties of sensor systems for measuring posture moments have been developed. To detect postural curvature variation of the spin, Wai Yin Wong and Man Sang Wong used accelerometers and gyroscopes [2]. A wearable-instrumented vest with three-axis accelerometers was produced for monitoring various postures [3]. To quantify human joint angle motion, C. Mattmann et al. employed conductive fiber sensors [4].

Lim et al. [5] created a wearable posture identification system to detect a correct sitting position by interfacing integrated accelerometers with the Arduino integrated development environment (IDE). However, the sensor only detected a single axis. Flexible resistive strain sensors were used to detect human motion [6]. Textile-based strain sensors have also been developed for use as wearables in monitoring physiological wellbeing [7]. Wearable strain sensors were designed by P. Tormene et al. and placed on biomedical clothing to quantify trunk range of motion [8]. A textile piezo-resistive pressure sensor was used to measure shoulder and neck movements [9], and for body-motion monitoring as well as touch and pressure sensors, textile-based devices with entirely polymeric conducting fibers were employed [10]. Dielectric elastomer sensors (DESs) were applied to measure all component motions of the wrist joints [11], and flexible resistive strain sensors made of polydimethylsiloxane were used to detect any type of human motion [12].

Numerous researchers have studied real-time posture tracking and monitoring. Minjeong Kim [13] developed a real-time monitoring sensor to detect human body sitting posture, and to help users assess and adjust their body balance. However, this sensor only detects the body position in the sitting position, and the current system design cannot monitor lateral bend or spine rotation. Ferdews Tlili et al. [14] studied a posture monitoring system composed of a smart belt equipped with inertial sensors. The sensors collect the posture information and send them to a cloud server via Wi-Fi connection. Human posture and walking speed monitoring using a wearable accelerometer sensor was studied by Wee-Soon [15]. However, the result showed that real-time monitoring was only functional at specific speed less than 10 km/h speed. C. Mattmann et al. employed conductive fiber sensors for monitoring elderly people’s postures and activities to facilitate healthy aging [16]. The research focused on the torso, covering the whole range of upper body postures. However, the reliability of the conductive track was not good. The thin metals could not withstand abrasion, especially during laundry action, so more solutions that are robust are required. Other research works [17] studied wireless wearable accelerometer sensors for activities, and detected walking and cycling events. P. Veske et al. developed and studied a stretchable circuit and its integration method on knit fabrics for lower back injury detection for cyclists. The conductive track was manufactured by using a TPU-coated rigid, but flexible, circuit. However, the studies were only concerned with comfort and reliability and did not include actual trunk monitoring [18]. Problems due to washing were also detected.

In this paper, a tight fitted garment and posture/orientation sensor was developed for measuring body posture. We focused on measuring the rider’s postural changes in terms of curvature variation of the spine and control trunk range of motion, and on detachable sensors to simplify the washing of the jersey. The main objectives of this study are: (1) developing a low-cost trunk-monitoring wearable sensor device to monitor posture during cycling and (2) detecting and controlling the real-time transmission of trunk movement data to enhance the system availability and allow immediate data analysis.

## 2. Materials and Methods

The methodological approach for this research contained two phases. The first phase dealt with the development and integration of sensor materials onto textile garments, while the second phase dealt with the measurement of real-time trunk orientation via sensors with integrated microcontrollers.

### 2.1. Developments of Sensor Materials

The developed smart garment consisted of two sensor modules, and a third module containing a digital data acquisition and feedback system and a battery pack. Each sensor module (size 21.2 mm length × 16.4 mm width × 3.3 mm height, and weight 2.1 g) consisted of an MPU6050 3-axis accelerometer and tri-axial gyroscope sensor orthogonally aligned and assembled on 3 mm laser cut rectangular MDF board with cutouts for magnets. They connect to a side pocket, where a removable Wemos ESP8266-based digital data acquisition unit (DAQ) is placed with its battery, programmed using the Arduino IDE. The sensors and microcontroller are then used in conjunction with textile electrodes and a textile conductive interconnecting circuit. All modules were attached to a stretchable tight-fitting garment via magnets.

#### 2.1.1. Design and Preparation of e-Textile Connection Lines

Four parallel conductive textile interconnection lines (42 cm length) were made, one from commercially available electrically conducting silver plated polyamide thread (CH-40, Madeira, Germany) [19], and one from Clevertex Hi-Cond CA 74 of VUB, CZ, a PES multifilament electro conductive hybrid thread [19]. In both cases, they were embroidered onto a 65% PA and 35% lycra knitted textile garment. These conductive tracks were embroidered on the fabric with a zigzag pattern, allowing easy stretch so that movements will not damage the conductive track. The linear resistance of the HC-40 was 288 Ω/m, while for CA 74 thread, this was 2.80 Ω/m.

#### 2.1.2. Circuit Design and Placement of the Sensor

**Figure 1:**
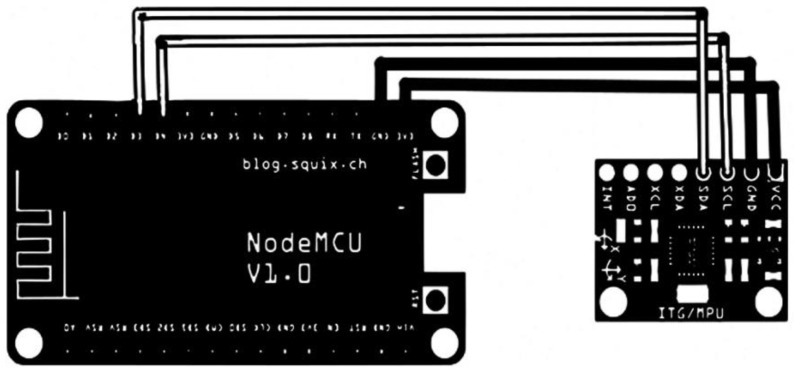
Circuit and schematic design MPU6050 accelerometer sensor with MCU ESP8266.

**Figure 2:**
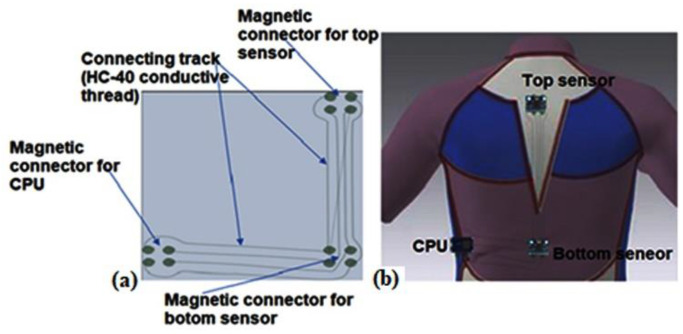
The e-textile interconnections line embroidered into the garment, with 4 encapsulated magnets per detachable module (**a**) and sensors attached on the t-shirt (**b**).

The sensor setup is seen in Figure 1. In this research, two measuring sensors were created on the prototype, one on the upper trunk and the other on the mid trunk (centrally) of the back (Figure 2). Each trunk sensor zone is fitted with MPU6050 gyro/accelerometer sensor modules. The central unit, a Wemos Node MCU ESP8266, is placed in a side pocket. This central unit, which regulates measurement transmission and data readout, is comprised of a microcontroller, Wi-Fi antenna, and a LiPo battery. The Wi-Fi antenna connects to an access point in the neighborhood, typically a hot spot created with a mobile phone. The MPU6050 is powered by a 3–5 V DC supply. The full-scale range for the detection of the angular velocity ranges from ±250 to ±2000°/s with a range of detectable acceleration in three dimensions from ±2 to ±16 g. The used accuracy level is selected through software; for our application ±250°/s and ±2 g is used.

A proper calibration that eliminates systematic errors and sensor biases is crucial for these MPU6050 gyro/accelerometer sensors to operate accurately. The techniques for calibrating accelerometers estimate a set of parameters using data from a variety of static orientations. These techniques are automatically performed using Arduino Uno software on the under static conditions at the flat surface. The measured acceleration should be proportional to the local gravitational acceleration. Finally, the systemic errors are already negligible and do not influence the output results.

#### 2.1.3. Connection Considerations

The connection of the rigid sensors on to the conductive track line was performed using a magnetic connector, which is embedded onto the fabric by embroidery. A magnetic connection can be used for the detachable sensor for wearable electronics. However, it can easily broken when the cyclist makes fast hard movements. In the current setup, in several tests, the connection between the control unit and one of the sensors was lost. These problems happen due to the jersey being highly elastic, leading to the magnet positions in the jersey shifting and no longer fitting well with the magnets mounted on the rigid MDF part. Therefore, further improvements in the magnetic strength and weight of the sensor should be considered in future iterations.

#### 2.1.4. Garment and Integration with Electronics

The accelerometers and gyroscopes integrated into the garment, which can detect postural changes (angle of bending) in terms of the curvature variation of the spine, were developed with the goal of improving the correct movement of the wearer. A system with coupling textile electrodes running into a channel with a meandering path between the two sensors was utilized for the interconnections between the sensors and central unit to allow unrestricted trunk movement while reducing mechanical strain on the interconnections; see Figure 2.

The interconnections were embroidered in a zigzag manner in the garment. A method of embedding magnets into the garment through embroidery was used to create the electrodes to which the MDF boards with the MPU6050 sensor, or the DAQ, must be attached.

### 2.2. Experimental Setup/Procedure

#### 2.2.1. Effects of Washing on the Electrical Resistance of the Conductive Truck

The conductive track was subjected to a washing process to assess its long-term durability properties against frequent washing. The sample was washed 17 times in a domestic washing machine at 30 °C using a conventional cleaning solution. After each washing cycle, the electrical linear resistance of the lines was measured using a Fluke 87 V digital multimeter in dry conditions. Two different sets of samples were prepared to determine the optimal integration technique of the sensors on the garment. On each sample, four different lines were tested, and the linear electrical resistance was recorded. These samples were washed 17 times at 30 °C in a washing machine.

#### 2.2.2. Evaluation of Garment Functionality and Usability

The measurements of real-time trunk monitoring were performed on a person at stationary and dynamic positions during the cycling activity. Stationary was sitting straight on the bicycle, making no movements. When a cyclist rides a bicycle with and without pedaling movements, the body is normally turning or tilting in different directions with reference to the ground. The integrated trunk sensor measured the real-time body position via the top and bottom sensors. In order to increase the accuracy of the measurement value, the gyroscope and the accelerometer sensor were initially calibrated using software based on the preliminary field test. Five different tests were conducted, and the average result was taken for analysis purposes with outside environments of 22 +/− 2 °C and 65 +/− 3% RH.

**Figure 3:**
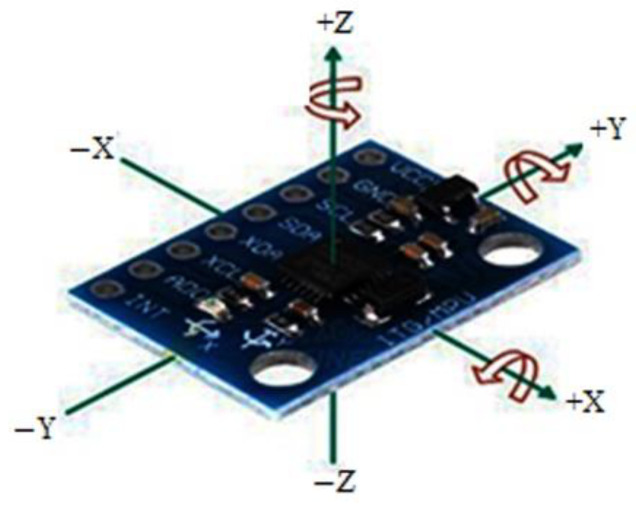
MPU6050 orientation and polarity rotation.

Next, the prototype outfit was tested on human subjects while they were cycling. The sensor modules, with measurement tolerances of ±3%, were assessed using the sensor orientation. As a result, the MPU6050 and ESP8266 can determine the tilt or trunk bending angle or rate of change of the angular position over time along the *X*, *Y*, and *Z*-axis (Figure 3). The MPU6050 is connected to the internet via the ESP8266, and at 50 Hz, the tilt angle data are sent to the Blynk cloud platform where they can be consulted. As a result, the back position can be monitored via this IoT technology. The angular velocity obtained must still be integrated to determine the angular position. When the bike began to move in a non-uniform motion, both the top and bottom gyro/accelerometer sensors tilted, leading to well-defined measurements in the three-dimensional (*X*, *Y*, *Z*) plane. As an outcome, the sensors’ positions changed. Furthermore, the top and bottom gyro/accelerometer sensors’ angular velocities were determined by measuring the angular position with change in time. Equation (1) can be used to compute the angular velocity:

(1) $$\omega =\frac{\;\Delta \theta \;}{\Delta t}$$

where ω refers to the angular velocity of the sensors, Δθ is the angular rotation and Δt is the change in time.

Angular acceleration is a primary phenomenon on gyro/accelerometer sensors in non-uniform motion in the three-dimensional (*X*, *Y*, *Z*) plane, in addition to the angular velocity. The angular acceleration occurred as the direction of the gyro/accelerometer changed, and it changed over time. Equation (2) was used to compute the angular acceleration:

(2) $$\alpha =\frac{\;\Delta \omega \;}{\Delta t}$$

Here, α denotes the angular acceleration, Δ*ω* is the change in angular velocity, and Δ*t* is the change in time.

The alignment of the three-axis accelerometer sensors in relation to one another is another crucial component of the sensor system. As a result, it is essential to assure the magnitude of the resultant acceleration in order to determine the tilt or trunk-bending angle between the top and bottom gyro/accelerometers. The relative angle between them can be calculated approximately from the magnitude of acceleration measured. Assume vector $U=(X_{1},Y_{1},Z_{1})$ to be the acceleration measured by the top sensor relative to its axis frame, and likewise $V=\left(X_{2},Y_{2},Z_{2}\;\right)$, the acceleration of the bottom sensor relative to its axis frame. Equation (3) [10] can be used to calculate the tilt angle (θ) between the two sensors, assuming all acceleration is, in reality, equal on both sensors relative to an outside frame of reference, which is a valid assumption in most circumstances for a cyclist:

(3) $$\theta_{\mathrm{rad}}=\mathrm{arc}\cos\left(\frac{\;U.V}{\left|\left|U\right|\right|\times \left|\left|V\right|\right|}\right)\;\mathrm{and}\;\;\theta_{\deg}=\theta_{\mathrm{rad}}\times \left(\frac{\;180}{\pi }\right).$$

## 3. Results and Discussion

### 3.1. Integration of the Orientation Sensor into Garment

**Figure 4:**
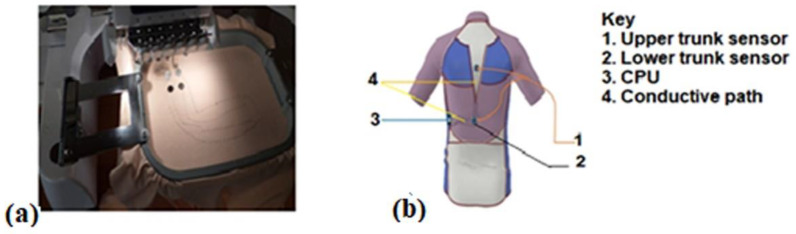
The embroidering process (**a**) and position of trunk monitoring sensors on to textile garments (**b**).

A knitted tight-fit shirt made out of fine-knitted fabric containing 60–75 percent polyamide and 25–40 percent elastomeric filaments in combination with ergonomic cuts was used for this application (Figure 4). The researchers’ past work was used to design and develop this shirt [20]. The purpose of this shirt is to provide support and restrict/limit the user’s range of motion on the back muscles in order to maintain the spine’s natural curvature when riding.

In the next step, the exact location of the sensor on the shirt was marked. The accelerometer sensors were placed on the upper and mid trunk. Subsequently, the sensor was attached to the shirt via magnetism, with the magnets embroidered into the shirt. Then, a conductive path to connect the microcontroller with the sensor was put on the shirt via embroidering (Figure 4a). The elongation percentage of CA-74 allows it to be used for human back posture determination because the elongation percentage is greater than 10%. Nevertheless, to make sure that the conductive path does not change the feel of the elastic fabric, the stitch was applied in a zigzag trajectory. Backing paper was attached under the knitting t-shirt to protect the knitted elastic t-shirt from sliding and stretching during the embroidery process. When the embroidery process was finished, this backing paper was removed.

In addition, the sensors and central unit were attached by glue on to a small rectangular MDF plate, which is on the shirt side and protects the electronic components from sweat. Next, conductive wire connections were made from the four embedded magnets. There were also four magnets embroidered in each electrode at the sensor locations and in the side pocket for the central unit. The location of the magnets on the shirt is based on the size when worn (around 10% stretch), so they match up with the fixed MDF plate magnets. With this setup, all components can be removed before washing. Note that in future versions, the components can be further miniaturized and embedded in a plastic enclosure so that they are fully protected from the outside environment (rain, snow).

### 3.2. Electrical Resistance Measurements of Thread-Washing Test

**Figure 5:**
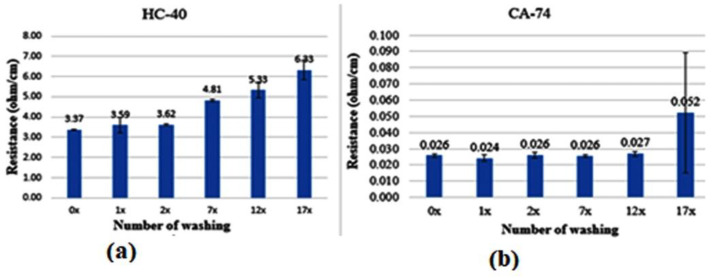
Electrical resistance of embroidered thread HC-40 (**a**) and CA-74 (**b**).

The washability tests for both HC-40 and CA-74 conductive yarns using domestic laundry washing are investigated in Figure 5.

Figure 5 illustrates the electrical resistance results of the washing tests of the two threads. The results of thread HC-40 showed that electrical resistance increased *up to* 87% *from its original*. These results indicate that the silver coating on the thread surface degrades by the water/detergent. Surface cracks on the conductive yarns as well as deformation along the transmission lines may occur after washing. These lead to an increase in the electrical resistance or conductor line discontinuities. The electrical resistance of all samples was raised after each washing. This makes HC-40 unsuitable to be used in e-textile communication lines that will undergo frequent washes. For one, the I2C data protocol used needs low resistance over the length of the connector (max length advised at 30 cm with low resistance). Moreover, the MPU6050 needs a guaranteed input voltage of 2.375 V, allowing a maximum line resistance of 250 Ohm with the input voltage of 3.3 V that the ESP8266 provides.

On the other hand, thread CA-74 showed that washing several times in a conventional washing machine did not notably influence the conductivity properties (Figure 5b), with them remaining below 0.1 Ω/cm. One reason for this durability is that the thread is a hybrid thread, made from ultrafine metallic wires combined with synthetic filaments (polyester). We can conclude that CA-74 shows much lower linear electrical resistance in Ω/cm than HC-40 thread, and is less influenced by washing, as required by smart textile applications (i.e., textrodes and sensors stitched on textile substrate).

Additionally, there is a variable tendency of the washing cycle on the electrical resistance of HC-40 conductive thread, as shown in Figure 5. After each washing cycle, however, the electrical resistance of CA-74 conductive thread samples increased only slightly. From Table 1, it can be seen that the electrical resistance increased somewhat with each washing cycle and was statistically significant (*p* < 0.001) for CH-40, as determined by ANOVA with alpha = 0.05 and 6 groups (0, 1 2, 7, 10 and 17 wash cycles). The statistical results of CA-74 conductive threads, on the other hand, were not statistically significant (*p* = 0.419). Furthermore, for the HC-40 conductive thread, the linear interpolation value R2 = 0.81 showed that there was a positive correlation between the washing cycle and electrical resistance. This was due to the effects of laundering on the individual fibers, which caused movement and damage across the HC-40 conductive thread structures. On the other hand, the R2 = 0.07 for CA-74 conductive thread showed that there was only a very slight positive correlation between the washing cycle and change in electrical resistance. This indicated that the laundry action on electrical resistance of CA-74 conductive thread was very limited. Hence, CA-74 conductive threads performed much better than CH-40. Therefore, only CA-74 conductive threads are suitable for usage in a cycling jersey, and hence they were selected for developing the conductive track in the prototype.

### 3.3. Trunk Posture Measurement

The data extracted from the accelerometers and the gyroscopes were validated. The ability of the human motion analysis device in measuring human body movement was also tested. We used Equations (2) and (3) to calculate the trunk tilt angle in the 3D plane using accelerometer data. The accelerometer data were dynamically employed to calculate the angle between the two sensors, and relate to the back posture.

#### 3.3.1. Accelerometer Data Analysis

**Figure 6:**
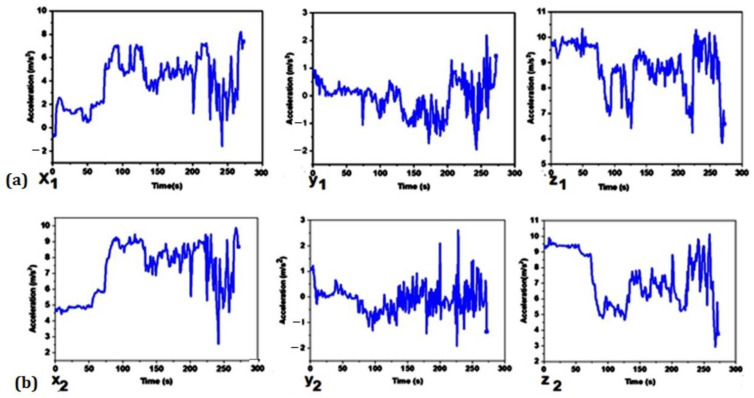
Top sensor (**a**) and bottom sensor (**b**) output acceleration for 3-axis accelerometer during 5 min of cycling.

The three axes (*X*, *Y*, and *Z*) moved in all directions and orientations, as the accelerometer and the shirt moved in all directions and orientations when riding a bike (Figure 6). Longitudinal acceleration was employed to detect a change in posture, and the location where the magnitude of the acceleration began to increase toward its highest peak was determined. In accordance, Figure 6a,b show both the movement of the top and bottom accelerometer sensor, respectively, during the dynamic posture of the subject. During the trial, the test subject was cycling for several minutes (approximately about 5 min).

The graph depicts various trends because of the cycling action. The typical data recorded during pedaling are presented, and both upper and lower trunk sensors show a predominant oscillation in all three axes. The acceleration readings were not consistent, indicating that varying acceleration occurred across the track. As the rider’s direction changed, so did the acceleration and deceleration. These occurrences can be identified as the speed signal and acceleration measurement in all three dimensions (*X*, *Y*, and *Z*) for both the upper and lower trunks. As a result of the findings, we may conclude that we can assess the trunk orientation and tilt angle of humans using this technology. The controller is designed for trainers and medical professionals to analyze trunk acceleration and deceleration. From the physical observation, it can be determined that when the cyclist bends to forward flexion or backward flexion from the flat position, the value of the acceleration in the *Z*-axis increases or decreases, respectively. For the trial given in Figure 6, from 0 to 72 s, the position of the cyclist was in normal forward bend over the steering wheel position, then 73 to 126 s, the cyclist sits up straight, while otherwise being in different types of flexion. In addition, when the cyclist was slightly turned to the left and right, the value of the acceleration in the *X*-axis was decreased, and vice versa. Comparing the top and bottom sensor, it is clear that they have a likewise trend, but there are clear differences, from which one can extract how much the back is bent.

In the *X* direction, there was a considerable difference in acceleration magnitude between the upper (top) and lower (bottom) sensors. The person was initially tilted in a forward flexion relative to the ground, and the *Z*-axis acceleration was almost the full gravitational acceleration (9.8 m/s^2^) for both sensors. More or less flexion switches the acceleration between *X* and *Z*, while the *Y* acceleration is the rotation of the torso, which is minimal during cycling, as observed through the *Y* acceleration values of the top and bottom sensors.

#### 3.3.2. Validation Results of Angular Velocity

**Figure 7:**
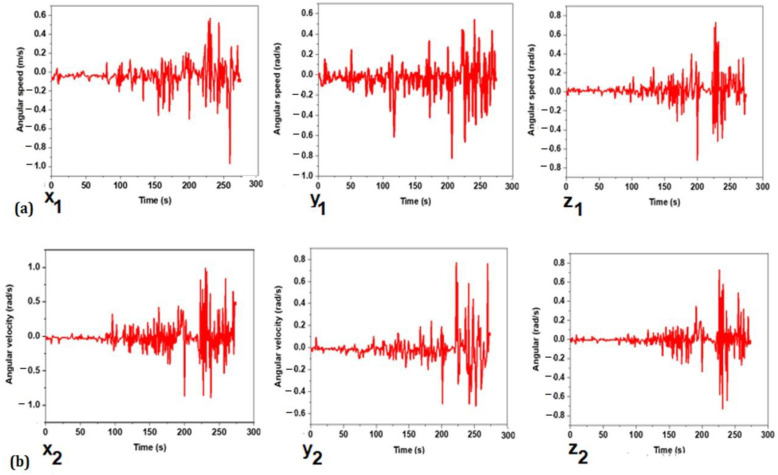
Top sensor (**a**) and bottom sensor (**b**) output angular velocity from the gyroscope.

The angular velocity values measured during the trial were plotted on a graph, and they reflect the amount of rotation that is being measured. Figure 7a,b show both the angular velocity of the top and bottom accelerometer sensors, respectively. The results of the measurement show that angular velocity increased with the rate of trunk rotation.

In addition, the cycling movement’s three-axis angular velocity varies substantially. Furthermore, it is possible to observe that the sensor placed on the upper trunk shows a lower rotational angle relative to the lower sensor. The rotation around the *Y*-axis behaves similarly between the two sensors. However, for the *X* and *Z* axes, there is a difference in the angular velocity observed at the top and bottom trunks. The angular velocity value at the bottom trunk is greater in the mean and pick-to-pick values than the upper trunk, indicating the cyclist can keep the upper torsion more immobile as compared to the lower trunk, which is closer to the legs performing the pedaling motion.

#### 3.3.3. Trunk Back Bending Angle

**Figure 8:**
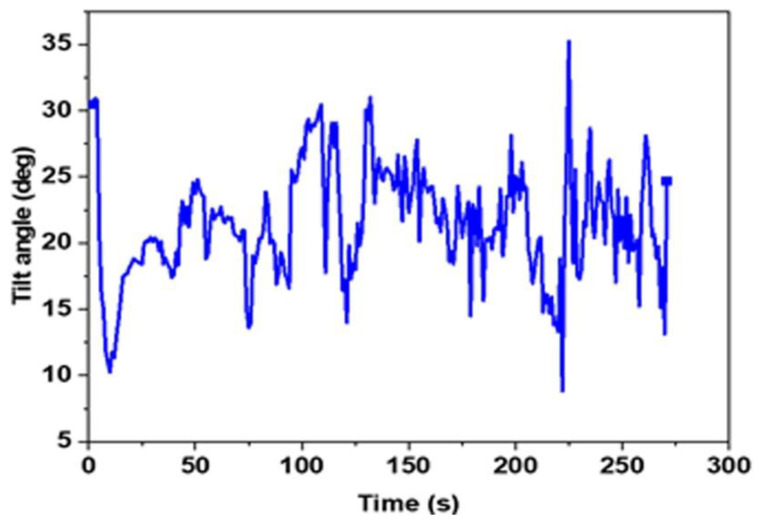
Trunk back bending angle.

The ideal position for road bike riders and mountain bikers varies from cyclist to cyclist. The ranges quoted for joint angles refers to the ‘Retul fit’ standard used in the former highly trained cyclists [21,22] The ‘angle of trunk’ inclination for cyclists is approximately 15°–30° to the world *Z*-axis relative to gravity. Equation (3) was used to calculate the trunk back bending angle, i.e., angle between the top and bottom accelerometer sensors. As the wearable motion sensor was placed close to the trunk via the tight-fitting shirt, measurement mistakes should be low. The trunk tilt angle as a function of time is depicted in Figure 8. The highest and lowest trunk tilt angles during cycling ranged from 8° at 222 s to 35° at 225 s, respectively.

According to the findings, the average trunk-bending angle was 21.5°, and 99 percent of the recorded angles was within the standard. This indicated that the cyclist was riding the bike in a normal position without bending the back excessively with reference to the ideal biking position. Furthermore, trainers can learn a lot about the route by monitoring how the angles vary as the race progresses. In addition, the cyclist also know how the effects of the trunk tilt angle influence the performance of the cyclist race [23,24].

## 4. Conclusions

To measure the tilt angle and monitor body postures and trunk movement, an innovative portable, user-friendly and wireless sensor network garment prototype was built, integrated with accelerometers and gyroscopes. This system measured the cyclist’s trunk tilt angle while cycling on the field and can be washed. The focus was on measuring the acceleration, angular velocity, and trunk tilt angle due to the importance of trunk dynamics in assessing cycling technique. It can be seen from the result that the system can capture live posture data. Specifically, in the test, 99% of the cyclist’s position was considered to be within the standards.

The integrated acceleration and gyroscope sensors on cycling outfits enabled authentic control of cyclist motion, avoiding potentially dangerous trunk movement that could result from trying to turn on a road, and maintaining the ability to ride comfortably in a normal posture, according to the developed system.

It is a huge step forward that such a little sensing system can assist cyclists during practice or racing. To extend the application of angular acceleration sensors, future work will aim at applying the developed sensor in the position monitoring and fault diagnosis of the position of the cyclist using textile-based actuators, which shows and immediately actuates when bad posture is detected. Further improvements in the connection method of the sensors to the jersey are also required to reduce the danger of the sensors coming loose.

For future work, we will investigate the variations in trunk posture on the sagittal and coronal planes during trunk movement. In addition, the incorporated sensors can be contained within a self-powering system by incorporated environmentally friendly conductive polymer composite super capacitors.

## Figures and Tables

**Table 1:** Overview of the statistical effects of washing on electrical conductivity.

	Source of Variation	SS	df	MS	F	*p*-Value	F Crit
HC-40	Between Groups	197.48	6	32.91	39.305	2.43 × 10^−18^	2.26
	Within Groups	46.89	56	0.837			
	Total	244.37	62				
CA-74	Between Groups	0.007	5	0.001	1.008	0.419	2.354
	Within Groups	0.093	66	0.001			
	Total	0.099	71				

## Data Availability

The data presented in this study are available on request from the corresponding author.
